# How legal awareness shapes developers’ data compliance behavior: A deterrence theory perspective

**DOI:** 10.1371/journal.pone.0356356

**Published:** 2026-08-17

**Authors:** Xinyuan Wang, Zhenyang Zhang

**Affiliations:** 1 School of Law and Economics and Management, Hulunbuir University, Hulunbuir, China; 2 Department of Law, Dongguk University, Seoul, Korea; A’Sharqiyah University, OMAN

## Abstract

As China’s digital regulatory framework continues to evolve, understanding what drives software developers’ data compliance behavior becomes increasingly important. Drawing on deterrence theory from a behavioral, law-in-action perspective, this study examines how data legal awareness influences data compliance behavior through perceived sanction certainty and perceived sanction severity, and how perceived compliance culture moderates these mediating pathways. Survey data collected from software developers were analyzed using structural equation modelling. The results indicate that data legal awareness promotes data compliance behavior directly and indirectly through perceived sanction certainty and perceived sanction severity, with certainty exerting a stronger mediating effect than severity. Perceived compliance culture significantly strengthens the effect of data legal awareness on perceived sanction certainty, while its moderating effect on perceived sanction severity receives only limited support. Theoretically, these findings extend deterrence theory to the data compliance domain by specifying the cognition–perception–behavior pathway and identifying the moderating role of compliance culture. Practically, the findings offer implications for how regulatory authorities allocate enforcement resources and how organizations design compliance management systems.

## 1. Introduction

In today’s era of flourishing global digital economy, data has emerged as the fifth major production factor following land, labor, capital, and technology, with its value creation profoundly reshaping the operational patterns of economy and society [1]. However, the large-scale flow and utilization of data have also brought unprecedented security risks. According to IBM’s 2023 report, the average cost of global data breach incidents reached $4.45 million, an all-time high. Facing the increasingly severe data security situation, countries worldwide have constructed comprehensive data protection legal systems. From the European Union’s General Data Protection Regulation (GDPR) to the California Consumer Privacy Act (CCPA), the world is entering an era of “strong regulation” in data compliance [2,3].

While this global tightening of data governance is evident, China presents a noteworthy case. As a major digital economy power with a rapidly expanding regulatory apparatus, China has adopted an increasingly systematic approach to data governance legislation. Since the implementation of the Cybersecurity Law in 2017, the subsequently enacted Data Security Law (DSL) (2021) and Personal Information Protection Law (PIPL, 2021) have collectively established a comprehensive legal architecture for data protection [4]. In terms of penalty ceilings, the PIPL stipulates fines of up to 50 million RMB or 5% of the previous year’s revenue, slightly exceeding the GDPR’s 4% threshold for data protection violations, while also introducing personal liability for directly responsible executives [5]. Beyond the magnitude of penalties, China’s enforcement regime is characterized by routine regulatory inspections of mobile applications, cross-agency coordination led by the Cyberspace Administration of China and other competent authorities, and public disclosure of violations, which collectively enhance the probability and visibility of detection [6]. This severity in penalties has brought significant compliance obligations to all participants in the Chinese market [4].

Against this backdrop, the role of software developers becomes particularly critical. As builders of digital products, their decisions in system design, coding, and data processing significantly influence whether enterprises can achieve the compliance requirements of Privacy by Design and Security by Default [7,8]. Recent research in a controlled lab study indicates that the majority of code submitted by developers is not fully compliant, and without external prompts or expert support, they tend to prioritize functionality and security over proactively embedding data protection, with few actively seeking compliance confirmation during the development process, illustrating the practical challenge of shifting from function-first to compliance-first [9]. Paradoxically, legal norms do not automatically translate into actual compliance behavior. Although developers hold the technical keys to compliance, they often lack systematic cognition and understanding of legal provisions, preferring to view compliance as a burden hindering innovation rather than a necessary guardrail [10].

Deterrence theory provides an important perspective for understanding this dilemma. Originating from the seminal work “On Crimes and Punishments” by 18th-century Italian jurist Cesare Beccaria, deterrence theory rests on the core assumption that individuals are rational decision-makers who weigh potential benefits against costs before acting. When individuals foresee that the punishment for non-compliant behavior exceeds potential benefits, they are more likely to refrain from such behavior [11]. In the information security field, deterrence theory has been widely applied to explain employee compliance behavior, but has mainly focused on adherence to internal organizational policies [12]. However, when the source of deterrence shifts from internal organizational norms to national laws, and when research subjects change from general employees to software developers who exercise technical control, the effectiveness of deterrence mechanisms warrants reassessment.

Although some existing studies have explored the impact of legal awareness or perceived deterrence on compliance behavior [13,14], significant research gaps remain. First, few empirical studies have systematically examined the psychological pathway from legal awareness to perceived sanctions, then to compliance behavior. Given China’s recently consolidated data governance framework centered on the Cybersecurity Law, Data Security Law, and PIPL, such investigation is both timely and necessary. Second, in China’s collectivist cultural context, individual behavior is deeply influenced by the organizational environment [15]. The compliance culture within organizations, understood as the shared values, beliefs, and behavioral norms regarding adherence to data protection laws and regulations, likely plays a crucial role in shaping deterrence effects, yet this mechanism remains underexplored.

Therefore, this study aims to address these gaps. We construct a moderated mediation model, using a sample of 351 Chinese software developers, to answer the following core questions: (1) Within the data protection regulatory framework described above, how does software developers’ legal awareness influence their data compliance behavior through the dual mediating effects of perceived sanction certainty and severity? (2) Can organizational-level perceived compliance culture moderate the relationship between legal awareness and perceived sanctions, thereby affecting overall deterrence effectiveness? (3) Do perceived sanction certainty and severity play differential roles in the mediation mechanism?

The theoretical contributions of this study are mainly reflected in three aspects: First and foremost, it provides contextual confirmation, within the domain of data governance and software development, that the effect of perceived sanction certainty exceeds that of perceived sanction severity. This pattern is consistent with prior deterrence research in other contexts, and the present study extends its validation to the data compliance domain. This finding reinforces deterrence theory by demonstrating that, within China’s recently strengthened regulatory framework, the likelihood of detection matters more than the harshness of penalties, thereby highlighting the psychological mechanism of certainty-driven compliance. Building on this core finding, by focusing on software developers as key actors, this study extends the application of deterrence theory to the developer level, providing new evidence on how legal awareness influences compliance behavior in the era of data‑intensive innovation. Additionally, it empirically tests an integrated mediation–moderation framework linking legal awareness, perceived sanctions, organizational compliance culture, and compliance behavior in a developing data-protection regulatory context. Specifically, the findings reveal that perceived compliance culture strengthens the positive relationship between legal awareness and perceived sanction certainty, demonstrating that organizational context can amplify individual‑level deterrence perceptions. This finding underscores the importance of incorporating organizational‑level factors into deterrence models, which have traditionally focused on individual cognition alone.

## 2. Literature review and hypotheses

### 2.1. Data legal awareness and compliance behavior

Legal awareness generally refers to an individual’s understanding and recognition of legal norms, as well as the extent to which they internalize their rights and obligations within the legal system. It encompasses both the static knowledge of legal provisions and a dynamic comprehension of legislative purposes, value orientations, and implementation effects [16–18]. As reviewed by Horák et al. (2021), legal awareness is a multidimensional construct encompassing general legal knowledge, situational understanding, attitudes toward the law, trust in legal institutions, and a sense of legal identity [19]. It therefore combines cognitive understanding of legal rules with value‑based orientations that can shape behavioral responses.

While legal awareness can thus encompass both normative and instrumental dimensions, in the context of data governance, this study focuses on its instrumental operation, which can be understood as a form of enforcement literacy that enables individuals to interpret and respond to legal requirements in light of potential detection and sanction [20]. Consistent with this reasoning, prior research [21,22] has demonstrated that a higher level of legal awareness enables individuals to anticipate legal risks, actively pursue compliant solutions, and thereby reduce the likelihood of violations at both decision‑making and operational levels. Conceptually, it operates at the individual level—reflecting a person’s internal cognition and self‑regulatory mindset, rather than an organization’s collective culture or institutional emphasis on compliance.

Data compliance refers to the concrete behavioral choices made throughout the entire data lifecycle—including collection, storage, processing, transmission, and deletion—in accordance with relevant laws, industry standards, and organizational requirements [23]. For instance, the collection of personal information requires organizations to inform data subjects of the purpose, scope, and retention period of data use. Data sharing and cross-border transfers typically necessitate compliance assessments or filing procedures. At the stage of data development and utilization, appropriate technical and managerial measures are expected to ensure data security.

From a theoretical perspective, legal awareness constitutes a fundamental cognitive precondition guiding compliance behavior [22]. According to normative compliance theory and rational choice theory, individuals with higher levels of legal awareness are more capable of recognizing the binding force of legal norms, perceiving potential legal risks and the associated sanctions, and thereby choosing compliant paths in cost–benefit analyses to protect their personal interests and social reputation [17]. In the domain of data governance, this proactive learning and normative interpretation driven by legal awareness enable individuals to embed compliance elements into data-processing procedures—for instance, classifying and protecting sensitive data, refining privacy policies and consent mechanisms, and establishing access-control and auditing systems [24].

Empirical research across related domains has generally supported the positive influence of legal awareness on compliance behavior. For example, Bulgurcu et al. (2010) found that information-security awareness significantly enhances employees’ compliance attitudes and behavioral intentions [25]. Building on this, Ifinedo (2012) demonstrated through the theory of planned behavior that legal knowledge, as a cognitive foundation, helps individuals form positive compliance attitudes by understanding the benefits of compliance and the costs of violations [26]. Within the field of environmental law, Chen et al. (2020) examined how citizens’ recognition of China’s new environmental law affects their pro-environmental behavior [13]. They found that for individuals with initially low compliance intentions, legal norms serve as an informative and guiding force, effectively shaping compliance willingness and promoting compliant behavior in daily practice.

While these findings provide converging evidence across domains, empirical research specifically examining software developers remains scarce, further motivating the present study. In the context of digital transformation, this theoretical linkage becomes particularly salient within certain professional groups. For software developers operating at the forefront of data-intensive application development, understanding and adhering to data-protection laws is increasingly regarded as a basic professional requirement. Developers with a higher level of legal awareness are more likely to translate such cognitions into concrete measures that ensure data compliance. Therefore, this study proposes the following hypothesis:

**H1.** Legal awareness has a significant positive effect on data compliance behavior.

### 2.2. Perceived certainty and severity of sanctions

#### 2.2.1. Deterrence theory.

General deterrence theory offers an essential lens for understanding how legal awareness translates into compliance behavior. Originating in criminology, this theory has been extensively applied in information systems security research to explain behaviors that either support or undermine information protection. Deterrence theory posits that individuals determine whether to comply with rules through a rational evaluation of the costs and benefits of violations, suggesting that sanctions that are certain, severe, and swift can effectively discourage deviant behavior [11]. Existing studies primarily emphasize two sanction dimensions—certainty and severity—while often excluding celerity, possibly because excessive focus on sanction swiftness may contradict prudential principles and procedural justice [27,28].

Specifically, perceived sanction certainty refers to an individual’s subjective probability judgment regarding whether their violations will be detected and punished by regulatory or law enforcement agencies; the higher this perceived likelihood, the stronger the certainty. In contrast, perceived sanction severity concerns an individual’s subjective evaluation of the intensity or magnitude of punishment they might face once violations are discovered; the greater the anticipated loss, the stronger the perceived severity [29].

#### 2.2.2. Data legal awareness and perceived sanctions.

Criminological research indicates that individuals typically possess a general understanding of legally prescribed sanctions, yet tend to show significant biases in estimating the likelihood and magnitude of these punishments [30]. According to information‑processing theory, individuals with higher levels of legal awareness—because of their more substantial legal knowledge reserves—can identify and assess violation risks more accurately [31,32]. Through randomized controlled experiments, Pickett et al. (2016) [33] found that when the legal information individuals receive contradicts their prior expectations, they significantly adjust their perception of sanction risks and increase uncertainty about potential consequences; this information‑updating process indirectly influences their willingness to commit unlawful acts.

Within a stringent data-protection legal framework, developers who are thoroughly familiar with these laws are better positioned to assess enforcement risks accurately. Similarly, D’Arcy et al. (2009) [11] demonstrated that employees’ awareness of security countermeasures enhances their perceived certainty that violations will be detected, thereby increasing their overall assessment of misuse risks. Accordingly, this study proposes the following hypotheses:

**H2a.** Legal awareness has a significant positive effect on perceived sanction certainty.

**H2b.** Legal awareness has a significant positive effect on perceived sanction severity.

#### 2.2.3. Perceived sanctions and data compliance behavior.

Building on the deterrence framework outlined above, a large body of empirical evidence supports the positive influence of perceived sanctions on compliance behavior. For instance, higher levels of perceived sanction certainty and severity have been found to significantly reduce users’ intentions to misuse information systems. Kuo et al. (2021) observed that when employees believe that violations may result in severe punishment [14], the perceived costs of misconduct substantially increase, leading them to prefer compliance when weighing the expected consequences of their actions.

Chen et al. (2012) further argued that the actual deterrent effect does not arise solely from the fear of punishment [34]. When employees decide whether to violate security policies, they engage in a comprehensive psychological evaluation that not only considers the expected certainty and severity of punishment but also incorporates incentive mechanisms and emotional factors such as anxiety or frustration. In their meta‑analysis, Trang & Brendel (2019) found that within information‑security policy compliance research [12], both sanction certainty and sanction severity are positively correlated with compliance behavior, though the effect of certainty tends to be more stable and stronger. This finding is consistent with classical deterrence theory predictions, which maintain that evidence favoring punishment certainty is more consistent across studies and that certainty exerts a stronger deterrent force than severity [35]. In summary, these studies indicate that individuals’ compliance decisions stem from rational evaluations of perceived sanction risks rather than from emotional fear alone. In the context of data governance, higher perceptions of sanction certainty and severity enhance awareness of regulatory consequences and encourage greater compliance intentions. Based on this, we propose:

**H3a.** Perceived sanction certainty has a significant positive effect on data compliance behavior.

**H3b.** Perceived sanction severity has a significant positive effect on data compliance behavior.

#### 2.2.4. The mediating role of perceived sanctions.

Building upon the preceding analysis, perceived sanction certainty and severity are proposed to play critical mediating roles between legal awareness and data compliance behavior. This mediating mechanism aligns with psychological reasoning about cognitive–perceptual–behavioral processes [36]. Specifically, legal awareness serves as a cognitive prerequisite that heightens individuals’ perceptions of both the likelihood of detection and the severity of potential penalties. These elevated perceptions, in turn, shift the cost–benefit calculus toward compliance-oriented behaviors.

A number of studies provide empirical evidence supporting such mediation effects. Bulgurcu et al. (2010) demonstrated that information security awareness shapes compliance attitudes and behaviors through perceived benefits and costs of compliance and non-compliance [25], where the perceived cost of noncompliance can be understood as encompassing an individual’s perception of sanctions. D’Arcy et al. (2009) more directly examined this mechanism and revealed that users’ awareness of security countermeasures mitigates information-system misuse intentions by influencing perceived sanction certainty and severity [11]. Jaeger et al. (2021) further found that information security policy awareness affects employees’ compliance through multi-level sanction perceptions [37], and that the strength of this mediation effect varies depending on individuals’ baseline compliance intentions.

Drawing upon these findings, this study proposes the following hypotheses:

H4a: Perceived sanction certainty mediates the relationship between legal awareness and data compliance behavior.

H4b: Perceived sanction severity mediates the relationship between legal awareness and data compliance behavior.

### 2.3. The moderating role of perceived compliance culture

Organizational culture constitutes a crucial contextual factor that influences employee behavior. In the domain of information security, numerous studies have shown that a strong compliance culture not only shapes employees’ compliance-related cognition but also profoundly affects the conversion process between cognition and behavior [38,39]. Neo-institutional theory holds that organizations adopt compliance practices not solely for efficiency considerations but also to gain legitimacy by conforming to coercive, mimetic, and normative pressures [40]. Grounded in this perspective, compliance culture reflects the shared understanding among organizational members regarding compliance expectations, the organization’s commitment to compliance, and the degree to which compliance norms are embedded in daily routines [41]. Unlike the individual-level legal awareness discussed in Section 2.1, perceived compliance culture captures employees’ shared reading of the organizational environment rather than their personal knowledge of specific legal provisions. Accordingly, perceived compliance culture in this study is conceptualized as an organizational‑level environmental perception, referring to employees’ overall perceptions and experiences of their organization’s values, norms, and practical actions concerning data protection and regulatory compliance [42,43].

Prior studies often view organizational‑level factors as moderating conditions that influence how individual‑level perceptions translate into behaviors. For instance, Jain et al. (2013) confirmed that perceived organizational support moderates the relationship between organizational stressors and organizational citizenship behavior [44]. Moquin and Wakefield (2016) further argued that software compliance is not merely a technical or economic necessity but also a manifestation of legal and institutional culture [45]. Together, these studies suggest that employees’ perceptions of organizational-level factors, whether framed as organizational support, institutional culture, or compliance commitment, can shape the strength of individual-level cognitive–behavioral relationships, providing a theoretical basis for examining PCC as a moderator in the present study.

However, recent experimental evidence from Horstmann et al. (2025) [9] demonstrates that even when professional developers receive privacy prompts and expert support, their GDPR‑compliant implementation rate remains notably low. This suggests that knowledge provisions or external guidance alone are insufficient to drive compliance behaviors in the absence of strong organizational norms and enforcement expectations. Their findings imply that contextual factors beyond individual knowledge, such as organizational compliance culture, may be necessary to translate awareness into compliant practice.

Drawing on social proof theory, when individuals face uncertainty, they tend to infer appropriate behavior by observing the actions of others [46]. In organizations characterized by a strong compliance culture, employees are more likely to witness consistent examples of compliant behavior. These observable social cues can reinforce the internalization of compliance norms, thereby strengthening the linkage between legal awareness and perceived sanctions. We focus on the first stage of the mediation because compliance culture most directly shapes the interpretive environment in which legal knowledge is translated into risk perceptions, whereas the link from perceived sanctions to behavioral compliance is more consistently driven by individual-level cost–benefit reasoning. Based on this reasoning, we propose the following hypotheses:

H5a: Perceived compliance culture positively moderates the impact of legal awareness on perceived sanction certainty.

H5b: Perceived compliance culture positively moderates the impact of legal awareness on perceived sanction severity.

**Fig 1:**
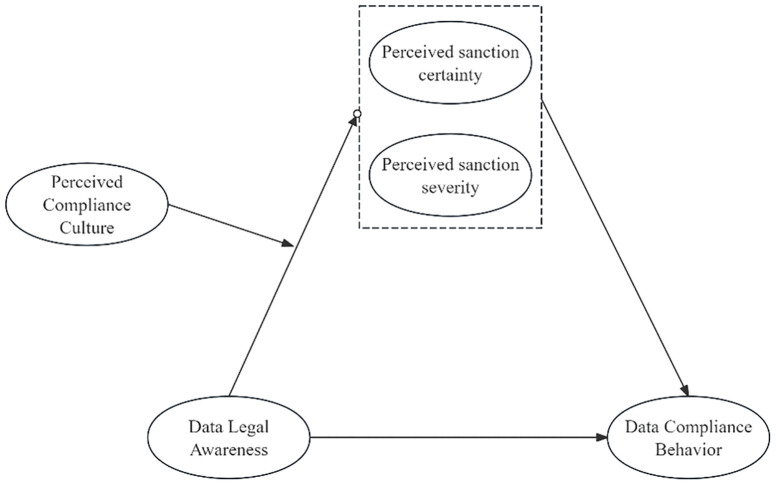
Research Framework.

The analysis framework of the research is shown in Fig 1 below.

## 3. Methods

### 3.1. Data collection

This study targeted software developers in China and employed a questionnaire survey method to collect data. In July 2025, questionnaires were distributed to two partner software development companies for pilot testing, yielding 43 valid responses. These 43 responses were used solely for instrument refinement and were excluded from the final dataset. The pilot testing served two purposes: first, to validate the applicability and clarity of the measurement scales, and second, to refine the operational definition of the target population. The results indicated that middle- and senior-level managers in the software companies frequently engage in hands-on development work alongside their managerial duties and were therefore included in the target population. The wording of several scale items was refined based on pilot feedback. During the formal survey phase, data were collected through the Credamo online survey platform between 20 August and 30 October 2025, with a target sample size of 400. After data cleaning and validity checks, 351 valid responses were retained for analysis. The survey was conducted anonymously in accordance with institutional ethics standards.

This study was reviewed and approved by the Institutional Review Board (IRB) of the School of Law and Economics and Management, Hulunbuir University (Approval No. 25062501). The project was classified as a low-risk management-science questionnaire study, and data collection through the third-party platform was authorized by the IRB. Electronic informed consent was obtained from all participants prior to participation.

Table 1 summarizes the demographic profile of the sample. The respondents were predominantly male (67.52%) and aged 25–34 (62.11%), with the majority holding a bachelor’s degree or above (82.05%). General developers accounted for 71.51% of the sample, with the remainder being middle or senior managers who also perform hands-on development work. Private enterprises represented 61.25% of the sample.

**Table 1:** Sample characteristics (N = 351).

	Category	Frequency	Percentage (%)
**Gender**	Male	237	67.52
	Female	114	32.48
**Age**	18-24	25	7.12
	25-34	218	62.11
	35-44	62	17.66
	45-54	46	13.11
**Education**	High school or below	5	1.42
	Junior college	58	16.52
	Bachelor’s degree	221	62.96
	Master’s degree or above	67	19.09
**Position**	Senior manager	19	5.41
	Middle manager	81	23.08
	General developer	251	71.51
**Ownership**	State-owned	136	38.75
	Private	215	61.25

### 3.2. Measures

Building upon existing research, the measurement instruments employed in this study were adapted and localized to reflect the specific characteristics of China’s data protection legal framework. All questionnaire items were measured using a 7-point Likert scale (1 = strongly disagree, 7 = strongly agree) to ensure precision and consistency.

Data Legal Awareness (DLA), as the independent variable, was adapted from prior studies [13,25], comprising 5 measurement items. This scale primarily assesses respondents’ level of awareness and depth of understanding regarding China’s data protection laws and regulations. A sample item includes “I fully understand the legal liabilities and sanction consequences that individuals and enterprises will face if they violate national data protection laws.”

Data Compliance Behavior (DCB), as the dependent variable, was adapted from prior studies [26,47], utilizing 5 measurement items. This scale aims to evaluate respondents’ behavioral performance in adhering to data protection standards in their actual work. A sample item is “I proactively comply with the organization’s internal data security management systems and operational procedures.”

Based on classic deterrence theory, this study conceptualizes deterrence perception as two distinct dimensions serving as mediating variables. Both dimensions were developed drawing from research by [11,27].

Perceived Sanction Certainty (PSC) is measured using 4 items. It assesses developers’ perception of the probability that non-compliant behavior will be detected. A sample item is “Given the current level of regulatory oversight, even minor data compliance oversights are likely to be detected.”

Perceived Sanction Severity (PSS) is measured using 4 items. It evaluates developers’ perception of the severity of consequences following non-compliance. A sample item is “If serious incidents occur due to improper data handling, responsible parties (including developers) may face legal liability.”

Perceived Compliance Culture (PCC), as the moderating variable, was primarily developed based on prior studies [47,48], comprising 5 measurement items. This scale aims to assess respondents’ perception of their organization’s data protection cultural climate. A sample item is “The organization provides us with adequate training to help us understand and apply data protection regulations.”

Five demographic variables- gender, age, education level, job position, and enterprise ownership type—were included as control variables to account for potential confounding effects on data legal awareness and data compliance behavior.

## 4. Results

### 4.1. Measurement model

SPSS 26.0 and AMOS 24.0 were used for data analysis. AMOS verified the measurement model, and mediation tests were performed with PROCESS in SPSS. This integrated approach is common in management and behavioral research [49]. Prior to confirmatory analysis, an exploratory factor analysis (EFA) was conducted to examine the underlying factor structure. The results indicated that the Kaiser-Meyer-Olkin (KMO) measure of sampling adequacy was 0.889, substantially exceeding the acceptable threshold of 0.6, and Bartlett’s test of sphericity reached significance (χ² = 3986.632, df = 253, p < 0.001), confirming the appropriateness of the data for factor analysis. The analysis extracted five factors with eigenvalues greater than 1, with a cumulative variance explained of 66.536% after rotation, confirming a clear five-factor structure.

As shown in Table 2, all constructs demonstrated Cronbach’s α coefficients exceeding 0.8, indicating good internal consistency reliability. The Average Variance Extracted (AVE) values for all five constructs surpassed the recommended threshold of 0.5, while Composite Reliability (CR) values all exceeded the standard requirement of 0.7. Furthermore, standardized factor loadings ranged from 0.596 to 0.879, with only two items slightly below the ideal standard of 0.7 but still above the acceptable standard of 0.5. Overall, these results demonstrate that the measurement model exhibits satisfactory internal consistency reliability and convergent validity.

**Table 2:** Reliability and validity analysis results.

	Items	Cronbach’s α	AVE	CR	Factor Loadings
**DLA**	5	0.856	0.548	0.858	0.645 ~ 0.781
**DCB**	5	0.831	0.530	0.833	0.596 ~ 0.776
**PSC**	4	0.853	0.609	0.862	0.760 ~ 0.795
**PSS**	4	0.848	0.585	0.849	0.726 ~ 0.829
**PCC**	5	0.885	0.614	0.888	0.719 ~ 0.879

Regarding discriminant validity assessment, Table 3 presents the comparison between inter-construct correlations and the square roots of AVE. According to the Fornell-Larcker criterion, the square root of AVE for each construct exceeded its correlations with other constructs, providing initial evidence of discriminant validity.

**Table 3:** Square roots of AVE and correlation coefficients matrix.

	M	SD	1	2	3	4	5
**1. DLA**	4.643	1.454	***0.740***				
**2. DCB**	4.878	1.429	0.426***	***0.728***			
**3. PSC**	4.821	1.547	0.443***	0.522***	***0.780***		
**4. PSS**	5.010	1.403	0.407***	0.442***	0.304***	***0.765***	
**5. PCC**	4.116	1.684	0.211***	0.148**	0.265***	0.222***	***0.783***

*Note:* The bold italic diagonal elements are the square roots of each AVE.

* p < 0.05; ** p < 0.01; *** p < 0.001.

Through confirmatory factor analysis (CFA), we compared the proposed five-factor model with several competing nested models. As shown in Table 4, the five-factor model demonstrated superior fit indices compared to alternative models. Specifically, the five-factor model yielded satisfactory fit statistics (χ²/df = 1.370, RMSEA = 0.033, CFI = 0.979, NFI = 0.926, TLI = 0.976, SRMR = 0.036), meeting the recommended thresholds for good model fit. In contrast, the four-factor, three-factor, two-factor, and single-factor models all exhibited substantially worse fit indices, further confirming good discriminant validity among the constructs.

**Table 4:** Overall fit indices of the measurement model.

	χ^2^/df	RMSEA	CFI	NFI	TLI	SRMR
**Five-factor** **(DLA, DCB, PSC, PSS, PCC)**	1.370	0.033	0.979	0.926	0.976	0.036
**Four-factor** **(DLA, DCB, PSC + PSS, PCC)**	3.571	0.086	0.850	0.805	0.831	0.075
**Three-factor** **(DLA + PCC, PSC + PSS, DCB)**	7.237	0.133	0.632	0.599	0.590	0.125
**Two-factor** **(DLA + PSC + PSS, DCB + PCC)**	7.970	0.141	0.585	0.555	0.541	0.174
**Single-factor** **(DLA + DCB + PSC + PSS + PCC)**	9.398	0.155	0.498	0.472	0.447	0.137
**Criteria**	<3	<0.08	>0.9	>0.9	>0.9	<0.08

Since all variables were self-reported at a single point in time, common method variance (CMV) may be a concern [50,51]. Several procedures were employed to assess this issue. First, Harman’s single-factor test was conducted through exploratory factor analysis (EFA). The unrotated results indicated that the first factor accounted for only 31.350% of the total variance, well below the 50% threshold, suggesting that no single factor dominated the variance structure. Second, as shown in Table 4, the hypothesized five-factor model demonstrated significantly better fit than all alternative models, further suggesting that the data structure was not driven by a common method artifact. Third, an unmeasured latent method construct (ULMC) was added to the five-factor CFA model and allowed to load on all observed indicators [50]. The changes in fit indices between the baseline five-factor model and the ULMC model were minimal: ΔCFI = 0.009, ΔTLI = 0.008, and ΔRMSEA = 0.007, all below the recommended thresholds [52,53]. These results suggest that CMV is unlikely to pose a serious threat to the findings.

### 4.2. Hypotheses test

As shown in Table 3, the correlation analysis results indicated that DLA was significantly and positively correlated with DCB (r = 0.426, p < 0.001), PSC (r = 0.443, p < 0.001), and PSS (r = 0.407, p < 0.001). PSC was significantly and positively correlated with DCB (r = 0.522, p < 0.001), as was PSS with DCB (r = 0.442, p < 0.001), providing preliminary validation for the study’s foundational hypotheses.

To test the main and mediation effects, this study constructed four regression models. Except for demographic control variables, all variables were z-standardized to reduce potential issues such as multicollinearity. As shown in Table 5, Model 1 indicated that DLA had a significant positive effect on DCB (β = 0.421, t = 8.607, p < 0.001), supporting H1.

**Table 5:** Regression analysis results.

	Model 1. DCB			Model 2. PSC			Model 3. PSS			Model 4. DCB		
	β	SE	t	β	SE	t	β	SE	t	β	SE	t
**Gender**	−0.137	0.105	−1.309	−0.064	0.103	−0.618	−0.103	0.106	−0.975	−0.086	0.092	−0.936
**Age**	−0.010	0.061	−0.156	−0.116	0.060	−1.927	0.052	0.062	0.848	0.020	0.054	0.370
**Education**	0.092	0.076	1.206	0.051	0.075	0.678	−0.005	0.077	−0.068	0.074	0.067	1.112
**Position**	0.001	0.084	0.008	0.089	0.083	1.072	−0.075	0.085	−0.879	−0.013	0.074	−0.172
**Ownership**	0.063	0.100	0.633	0.123	0.099	1.247	0.052	0.101	0.513	0.004	0.088	0.043
**DLA**	0.421***	0.049	8.607	0.442***	0.048	9.178	0.402***	0.049	8.124	0.150**	0.050	2.981
**PSC**										0.373***	0.049	7.693
**PSS**										0.264***	0.047	5.575
**R2**	0.189			0.213			0.172			0.382		
**Adjusted** **R** ^**2**^	0.175			0.199			0.157			0.367		
**F**	13.399***			15.493***			11.878***			26.393***		

Note: * p < 0.05; ** p < 0.01; *** p < 0.001.

In Models 2 and 3, DLA exerted significant positive effects on PSC (β = 0.442, t = 9.178, p < 0.001) and PSS (β = 0.402, t = 8.124, p < 0.001), respectively, thus supporting H2a and H2b. Model 4 further showed that both PSC (β = 0.373, t = 7.693, p < 0.001) and PSS (β = 0.264, t = 5.575, p < 0.001) significantly predicted DCB, supporting H3a and H3b. After including the mediating variables, the effect of DLA on DCB was reduced but remained significant (β = 0.150, t = 2.981, p < 0.01), indicating potential mediation effects.

#### 4.2.1. Mediation effect analysis.

We further employed bias-corrected bootstrap methods with 5,000 resamples and a 95% confidence interval to test the mediation effects of PSC and PSS. The results showed that the total effect of DLA on DCB was significant (β = 0.421, p < 0.001, 95% CI [0.325, 0.517]). This coefficient indicates a moderate‑to‑strong positive association, suggesting that employees’ legal awareness exerts a meaningful influence on their compliance behavior. After including the mediating variables, the direct effect of DLA on DCB remained significant (β = 0.150, p < 0.01, 95% CI [0.051, 0.248]), indicating partial mediation. Specifically, the indirect effect of DLA on DCB through PSC was β = 0.165 (95% CI [0.121, 0.218]), and the indirect effect through PSS was β = 0.106 (95% CI [0.064, 0.160]). Since the 95% confidence intervals for both indirect effects did not include zero, PSC and PSS played significant mediating roles in the relationship between DLA and DCB. The total indirect effect was β = 0.271 (95% CI [0.211, 0.337]), supporting H4a and H4b. This indicates that about the majority of the total effect of DLA on DCB operates through the mediating mechanisms. Taken together, these findings suggest that employees’ legal awareness promotes compliance primarily by heightening their perceptions of sanction certainty and severity, underscoring the pivotal role of sanction‑based cognition in translating awareness into behavior.

#### 4.2.2. Moderation effect analysis.

Building on the confirmed mediation effects, this study further employed Process macro (Model 7) to test the moderating role of PCC in the relationships between DLA and the two mediating variables. As shown in Table 6, DLA demonstrated significant effects on DCB, PSC, and PSS. The interaction term between DLA and PCC had a significant effect on PSC (β = 0.270, t = 5.714, p < 0.001), but was also significant, though with a notably smaller coefficient, on PSS (β = 0.122, t = 2.405, p < 0.05).

**Table 6:** Moderation effect analysis results.

	DCB			PSC			PSS		
	β	SE	t	β	SE	t	β	SE	t
**Gender**	−0.086	0.092	−0.936	−0.068	0.097	−0.701	−0.102	0.104	−0.983
**Age**	0.020	0.054	0.370	−0.114	0.056	−2.025	0.056	0.061	0.921
**Education**	0.074	0.067	1.112	0.024	0.070	0.339	−0.020	0.076	−0.267
**Position**	−0.013	0.074	−0.172	0.117	0.078	1.497	−0.055	0.084	−0.659
**Ownership**	0.004	0.088	0.043	0.115	0.093	1.236	0.058	0.100	0.580
**DLA**	0.150**	0.050	2.981	0.391***	0.046	8.450	0.366***	0.050	7.365
**PCC**				0.161***	0.046	3.467	0.134**	0.050	2.685
**DLA × PCC**				0.270***	0.047	5.714	0.122*	0.051	2.405
**PSC**	0.373***	0.049	7.693						
**PSS**	0.264***	0.047	5.575						
**R** ^**2**^	0.382			0.311			0.205		
**Adjusted** **R** ^**2**^	0.367			0.293			0.184		
**F**	26.393***			19.279***			11.012***		

Note: * p < 0.05; ** p < 0.01; *** p < 0.001.

As shown in Table 7, for the PSC pathway, under low-level conditions (M-1SD), the Bootstrap 95% CI included zero (CI: [−0.003, 0.104]), while under mean-level (β = 0.146, CI: [0.103, 0.201]) and high-level conditions (M + 1SD, β = 0.246, CI: [0.177, 0.327]), the Bootstrap 95% CIs did not include zero. Furthermore, the index of moderated mediation for PSC was 0.101 (Bootstrap 95% CI: [0.062, 0.147]), which did not include zero, indicating that PCC had a significant moderated mediation effect in the relationship between DLA and PSC, supporting H5a.

**Table 7:** Conditional indirect effect and index of moderated mediation.

		Conditional Indirect Effect				Index of Moderated Mediation			
		Effect	BootSE	BootLLCI	BootULCI	Index	BootSE	BootLLCI	BootULCI
**PSC**	**M-1SD**	0.045	0.027	−0.003	0.104	0.101	0.022	0.062	0.147
	**M**	0.146	0.025	0.103	0.201				
	**M + 1SD**	0.246	0.038	0.177	0.327				
**PSS**	**M-1SD**	0.064	0.025	0.023	0.122	0.032	0.016	0.006	0.070
	**M**	0.096	0.024	0.056	0.150				
	**M + 1SD**	0.129	0.032	0.075	0.200				

However, for the PSS pathway, although the index of moderated mediation was statistically significant (index = 0.032, Bootstrap 95% CI [0.006, 0.070]), the conditional indirect effects of PSS remained significant across all conditional levels of PCC (low [M – 1 SD], mean, and high [M + 1 SD]). Specifically, the Bootstrap 95% confidence intervals for all three conditions did not include zero, suggesting that while the moderated mediation effect was significant, it was relatively weak in magnitude. These results indicate that PCC exerted a modest moderating influence on the indirect relationship between DLA and DCB through PSS, thereby providing only limited support for H5b. Overall, these findings suggest that a stronger compliance culture amplifies the positive influence of legal awareness on developers’ perceived sanction certainty, thereby enhancing their compliance behavior.

## 5. Discussion

### 5.1. Discussion of key findings

Adopting a behavioral, law-in-action perspective rather than a doctrinal legal analysis, this study draws on deterrence theory and compliance culture theory to investigate how developers’ understanding of data protection laws shapes their compliance behavior through perceived sanctions, and how a strong compliance culture reinforces these effects. The empirical analysis centers on four key constructs, namely data legal awareness, perceived sanction certainty, perceived sanction severity, and perceived compliance culture, to explain how legal understanding influences risk perception and subsequent compliance actions. Overall, the findings indicate that legal awareness encourages developers to evaluate the risks of non-compliance more carefully and to act in accordance with regulatory and organizational requirements.

First, data legal awareness exerts a significant positive effect on data compliance behavior (supporting H1). In this study, legal awareness is operationalized as an instrumental cognition, specifically a form of enforcement literacy that reflects developers’ knowledge of applicable data protection laws, regulatory obligations, and the legal consequences attached to non-compliance. This finding suggests that when software developers possess a clearer understanding of their legal obligations and accountability, they are more inclined to proactively comply with relevant regulations and organizational policies. Legal knowledge thus functions not only as an external constraint but also as a cognitive resource that shapes compliance-relevant decision-making, consistent with deterrence theory’s core proposition that awareness of legal consequences guides behavior.

Second, data legal awareness significantly enhances developers’ perceptions of both sanction certainty and sanction severity (supporting H2a and H2b), implying that individuals with greater legal awareness are more cognizant of the risks and consequences of violations. Both perceived sanction certainty and perceived sanction severity significantly promote compliance behavior (supporting H3a and H3b), consistent with the core proposition of deterrence theory that individuals are more likely to comply when they believe violations will be detected and punished.

An important empirical finding, however, is the consistent predominance of sanction certainty over sanction severity. This pattern aligns with classical deterrence theory, which argues that certainty is the stronger deterrent because it activates both legal and extralegal consequences such as reputational or social sanctions [54]. Recent evidence supports this dynamic: Gómez-Bellvís et al. (2025) show that when detection certainty is already high, increasing punishment severity yields diminishing marginal returns [55]. This pattern also departs from the classical information security policy compliance model proposed by D’Arcy et al. (2009), which emphasized sanction severity within internal organizational deterrence structures. Our findings suggest that in externally regulated and technologically auditable contexts, such as data governance, the salience of detection probability becomes a more decisive compliance trigger. This dominance of certainty may reflect developers’ professional rationality: as technically skilled actors whose work tends to be highly auditable through systematic peer review and contribution tracking [56] and closely tied to professional reputation [57], they may be particularly sensitive to the perceived probability of detection rather than to abstract threats of punishment severity.

Third, perceived sanction certainty and perceived sanction severity partially mediate the relationship between data legal awareness and data compliance behavior (supporting H4a and H4b). This suggests that legal awareness indirectly promotes compliance behavior by activating risk perception mechanisms while also exerting a direct influence. This mediating pattern aligns with the findings of Bulgurcu et al. (2010) [25]. Consistent with the certainty-severity pattern discussed above, the indirect effect through perceived sanction certainty is stronger than that through perceived sanction severity. One contextual explanation is that China’s data governance framework, which emphasizes regulatory oversight mechanisms such as compliance audits and administrative inspections under the PIPL and DSL, heightens developers’ sensitivity to detection probability, whereas the relatively uneven application of severe penalties across different legislative instruments may weaken their deterrent impact.

Moreover, compliance culture shows a significant positive moderating effect on the relationship between data legal awareness and perceived sanction certainty (supporting H5a), whereas its moderating effect on the relationship between data legal awareness and perceived sanction severity receives only limited support (H5b). Although the moderation index for H5b was statistically significant, the effect magnitude was small and the conditional indirect effects differed only slightly across levels of compliance culture, indicating limited substantive impact. Thus, compliance culture appears to strengthen the link between legal awareness and perceived detection likelihood, but contributes only modestly to shaping perceptions of punishment severity. In organizations with a strong compliance climate, employees are more likely to regard legal responsibility as a shared organizational norm, which amplifies the effect of legal awareness on their assessment of detection probability. This finding is consistent with D’Arcy and Greene (2014) [38], who argued that an active compliance or security culture promotes recognition of sanction mechanisms and accountability norms, thereby enhancing overall deterrent effectiveness.

Overall, the findings validate the applicability of deterrence theory in the data compliance context and reveal a clear mediation pathway, running from legal awareness through sanction perceptions to compliance behavior, within China’s rapidly developing data governance landscape. While the directional effects are generally consistent with previous information security compliance research [25,26], the effect magnitudes and the relative dominance of sanction certainty may partly reflect institutional features of China’s current regulatory framework. Specifically, recent legislative instruments such as the PIPL and DSL have established regulatory inspections, cross-agency information sharing, and public disclosure of violations, which collectively increase the perceived inevitability of detection. Public disclosure further creates reputational spillover effects that reinforce certainty-based deterrence.

### 5.2. Practical and theoretical implications

#### 5.2.1. Theoretical implications.

First, this study extends deterrence theory to the field of data compliance within China’s evolving data governance context. The results confirm clear links among data legal awareness, perceived sanction, and data compliance behavior, demonstrating that deterrence theory can explain developers’ compliance decisions in organizational settings. Importantly, the findings provide contextual confirmation of the deterrence principle that certainty of detection matters more than severity of punishment. While this pattern has been established in criminology and information security research, the present study extends its applicability to the domain of data governance and software development, where the auditable nature of development workflows and the reputational salience of code quality may amplify developers’ sensitivity to detection probability.

Second, by including perceived compliance culture as a moderating factor, this study broadens the scope of deterrence theory and adds an organizational normative dimension to its framework. Traditional deterrence models focus mainly on individuals’ rational assessment of sanction certainty and severity. Our findings show that a strong organizational compliance climate strengthens the influence of data legal awareness on perceived sanction certainty, although its moderating effect on perceived sanction severity receives only limited support. This finding reinforces the argument that deterrence operates not only through individual rational calculation but also through the normative environment in which individuals are embedded, and extends this perspective to the data compliance domain.

Third, by demonstrating that perceived sanction certainty and severity partially mediate the relationship between data legal awareness and compliance behavior, this study unpacks the internal transmission mechanism of deterrence in the data compliance context. Legal awareness not only directly promotes compliance but also activates risk perceptions that further drive behavioral conformity. This mediation evidence moves beyond testing whether deterrence works to explaining how it works, offering a more nuanced understanding of the compliance decision-making process.

#### 5.2.2. Practical implications.

First, enterprises and managers should treat data compliance as an essential part of corporate governance. Regular training on data protection laws can enhance developers’ data legal awareness and understanding of potential risks, thereby improving their ability to handle compliance issues in practice. Since this study finds that compliance culture strengthens the effect of legal awareness on perceived sanction certainty, management should institutionalize compliance as a shared organizational norm through concrete measures such as regular compliance communication, visible leadership commitment, and incorporation of compliance performance into evaluation criteria.

Second, governments and regulators should prioritize strengthening detection capabilities and ensuring consistent enforcement, rather than relying primarily on raising penalty levels. At the same time, enforcement strategies should be guided by principles of procedural fairness, transparency, and proportionality. Enforcement procedures should be clearly communicated to regulated parties, penalties should be proportionate to the nature and severity of the violation, and developers and organizations should have access to clear regulatory guidance and fair appeal mechanisms. Clearer coordination among regulatory bodies can further enhance the credibility and consistency of enforcement.

Third, at the industry level, the moderating role of compliance culture identified in this study suggests that industry associations and cross-organizational bodies should promote the standardization of compliance norms. Initiatives such as industry-wide compliance codes, peer review mechanisms, and shared best-practice frameworks can help establish a collective normative environment that extends beyond individual organizations, complementing both organizational and regulatory deterrence mechanisms.

### 5.3. Limitations and future research directions

First, the data were collected through a cross-sectional, self-reported survey administered via an online platform. Although screening criteria were applied to ensure that respondents were practicing software developers, the non-random sampling strategy may limit external validity. The cross-sectional design precludes strong causal inference; while the hypothesized direction is grounded in deterrence theory, reverse causality cannot be fully excluded, as cross-sectional data alone cannot conclusively differentiate between competing causal directions. To empirically examine this concern, a competing structural model with reversed paths was estimated. Both the hypothesized and reversed models demonstrated acceptable fit, with a ΔAIC of 4.07, below the threshold of 7 recommended for conclusive model preference [58]. This result confirms that cross-sectional data cannot definitively distinguish between competing causal directions, and future research should adopt longitudinal or experimental designs to more rigorously establish causal ordering.

In addition, although multiple statistical remedies were applied to assess common method variance, bias inherent in single-source self-report data cannot be entirely eliminated. In particular, self-reported compliance behavior may be subject to social desirability bias, whereby respondents over-report their adherence to data protection regulations. Although procedural measures such as guaranteed anonymity and the absence of evaluative framing were employed to mitigate this tendency, they cannot fully eliminate it. Furthermore, while several demographic variables were controlled, firm-level factors such as company size, industry type, and regulatory audit exposure were not included due to data constraints. Future research could incorporate multi-source or objective behavioral data and examine firm-level influences to strengthen both causal inference and external validity.

Second, the current model focuses primarily on deterrence-related constructs. While this focus allows a clear test of the deterrence mechanism in the data compliance context, it leaves other potentially relevant factors unexplored. Future research could incorporate additional psychological or contextual variables, such as organizational trust, moral norms, or regulatory uncertainty, to extend the explanatory scope of the cognition–perception–behavior framework.

Third, the sample is drawn exclusively from the Chinese digital regulatory environment, where recent legislative developments and enforcement practices may shape how developers perceive legal deterrence and compliance culture in ways that differ from other jurisdictions. Future studies are encouraged to test the proposed model across different countries, industries, and cultural settings to examine its cross-contextual stability and generalizability.

## 6. Conclusion

This study drew on deterrence theory to develop and empirically test a model explaining how data legal awareness shapes data compliance behavior among software developers in China’s digital regulatory environment. The results demonstrate that data legal awareness promotes compliance behavior both directly and indirectly through perceived sanction certainty and perceived sanction severity, with certainty exerting a stronger deterrent effect than severity. Perceived compliance culture further strengthens the influence of legal awareness on perceived sanction certainty, indicating that the organizational normative environment plays a meaningful role in activating the deterrence mechanism. These findings extend deterrence theory to the data compliance domain by clarifying the cognition–perception–behavior transmission pathway and introducing compliance culture as a boundary condition, while offering actionable guidance for organizations and regulators to prioritize detection capability, procedural fairness, and institutionalized compliance norms. Although the cross-sectional and single-context design of this study warrants caution in generalization, the proposed framework provides a foundation for future research to examine these relationships across different regulatory environments, over time, and with broader theoretical integration.

## Supporting information

S1 File Dataset. (XLSX)
